# Development of a tool to improve the quality of decision making in atrial fibrillation

**DOI:** 10.1186/1472-6947-11-59

**Published:** 2011-10-06

**Authors:** Liana Fraenkel, Richard L Street, Terri R Fried

**Affiliations:** 1 Department of Medicine, Yale University School of Medicine, New Haven, CT, USA; 2 Clinical Epidemiology Research Center, VA Connecticut Healthcare System, West Haven, CT, USA; 3 Department of Communication, Texas A&M University, College Station, TX, USA; 4 The Houston Center for Quality of Care and Utilization Studies and Baylor College of Medicine; Houston, TX, USA

**Keywords:** non-valvular atrial fibrillation, decision support, comorbidity

## Abstract

**Background:**

Decision-making about appropriate therapy to reduce the stroke risk associated with non-valvular atrial fibrillation (NVAF) involves the consideration of trade-offs among the benefits, risks, and inconveniences of different treatment options. The objective of this paper is to describe the development of a decision support tool for NVAF based on the provision of individualized risk estimates for stroke and bleeding and on preparing patients to communicate with their physicians about their values and potential treatment options.

**Methods:**

We developed a tool based on the principles of the International Patient Decision Aids Standards. The tool focuses on the patient-physician dyad as the decision-making unit and emphasizes improving the interaction between the two. It is built on the recognition that the application of patient values to a specific treatment decision is complex and that the final treatment choice is best made through a process of patient-clinician communication.

**Results:**

The tool provides education incorporating patients ' illness perceptions to explain the relationship between NVAF and stroke, and then presents individualized risk estimates, derived using separate risk calculators for stroke and bleeding over a clinically meaningful time period (5 years) associated with no treatment, aspirin, and warfarin. Sequelae of both stroke and bleeding outcomes are also described. Patients are encouraged to verbalize how they value the incremental risks and benefits associated with each option and write down specific concerns to address with their physician. A physician prompt to encourage patients to discuss their opinions is included as part of the decision support tool. In pilot testing with 11 participants (mean age 78 ± 9 years, 64% with ≤ high-school education), 8 (72%) rated ease of completion as "very easy," and 9 (81%) rated amount of information as "just right."

**Conclusions:**

The risks and benefits of different treatment options for reduction of stroke in NVAF vary widely according to patients' comorbidities. This tool facilitates the provision of individualized outcome data and encourages patients to communicate with their physicians about these risks and benefits. Future studies will examine whether use of the tool is associated with improved quality of decision making.

## Background

Nonvalvular atrial fibrillation (NVAF) is associated with an increased risk of stroke. Several randomized controlled trials have demonstrated a reduction in risk of future stroke with oral anticoagulants and antiplatelet agents, but at an increased risk of bleeding [1]. Decision-making about appropriate therapy to reduce this stroke risk involves the consideration of trade-offs among the benefits, risks, and inconveniences of different treatment options.

Guidelines addressing NVAF recommend the use of warfarin in patients with NVAF at high risk for stroke and aspirin for patients at lower risk [2,3]. However, both stroke and bleeding risk vary widely according to each patient's specific comorbidities [4,5]. Because of this variability in risk, there is a large range in the incremental risks and benefits associated with warfarin, aspirin and, no treatment [6]. Moreover, a review of studies examining patients' preferences regarding treatment for NVAF concluded that patient preferences frequently differ from treatment recommendations provided by guidelines [7]. Because of the variability in outcomes and preferences for the treatment of NVAF, the decision to initiate and maintain anticoagulation should ideally be based on a consideration of individualized outcome data and how patients value these outcomes [6].

Several decision aids have been developed to provide patients with information regarding risks and benefits of aspirin and warfarin and to help patients clarify their preferences regarding these options [8-10]. These aids, while stratifying patients' risk of stroke according to their comorbidities, have either not similarly stratified patients' risk of bleeding or have stratified bleeding risk for warfarin according to limited patient characteristics. In addition, existing decision aids have generally focused on the provision of information to patients. However, the provision of information, while necessary, is often not sufficient to improve the process of decision making [11]. To ensure informed choice, patients must be able be to effectively communicate their questions, concerns, and preferences to their physicians. In this paper we describe the development of comprehensive decision tool that focuses on preparing the patients to communicate with his/her physician to improve the quality of decision making in NVAF.

## Methods

**Figure 1:**
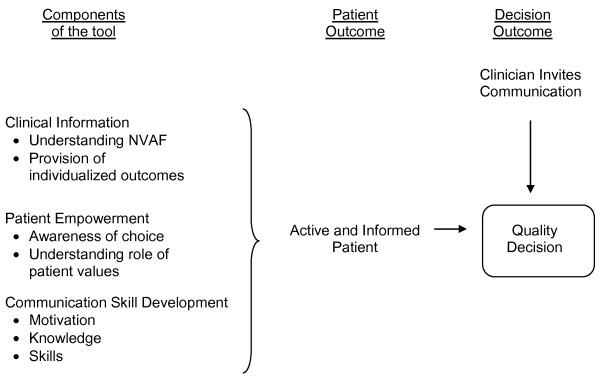
**Conceptual model guiding design of tool**.

The tool was designed to conform to the International Patient Decision Aids Standards (IPDAS) [12]. These criteria informed the process of tool development and the content, in terms of the description of treatment options, presentation of outcome probabilities, and helping patients to clarify their values. In addition to these criteria, development was guided by the conceptual model illustrated in Figure 1, which highlights the need to prepare patients to participate in the decision making process by helping them to understand the availability of options and the role of values and to communicate effectively with their clinicians, taking into account individual variability in preferred decision-making style.

## Results

Tool development occurred in an iterative process, in which the tool was administered to several participants, who were encouraged to ask questions and provide comments. The research assistant periodically assessed participants' understanding and recorded both participant feedback and her own observations, and these were used to make revisions to the tool. The revised version was then administered to the next group of participants, in order to elicit another round of feedback. This process continued until no additional clarifications were needed. This process led to several fundamental contributions to tool development: 1) participants found it difficult to understand how a problem in the heart could cause a problem in the brain. We therefore sought to create an educational component based on patients' illness perceptions; 2) in order to keep participants engaged, they required opportunities to think aloud about the material they were seeing and to interact with the research assistant.

The rational justifying each component is described in the following sections.

### Mode of Employment

The decision tool was designed to be used immediately prior to a regularly scheduled health maintenance appointment in a primary care setting, using a laptop computer and administered by a trained facilitator, using a script that is read word-for-word. Although the most appropriate time to implement decision support for NVAF may be at the time of diagnosis, in many settings the incidence of new NVAF is too low and the system barriers too numerous to introduce a decision support tool at the time of diagnosis. We therefore developed the tool to address prevalent NVAF, which allows the physician and patient to re-examine treatment decision-making as patients' risk factors for stroke and bleeding change over time.

#### Enhancing Understanding of NVAF

Effectively informing patients requires that patients have accurate illness perceptions about the cause of stroke in NVAF [13]. We therefore included the following educational component:

"Today we are going to be talking about atrial fibrillation, which I will be referring to as AFib. Afib is a heart problem. It's not the same heart problem that gives you a heart attack. Even though Afib is a heart problem it can cause a stroke. People generally think that heart diseases only cause problems in the chest."

**Figure 2:**
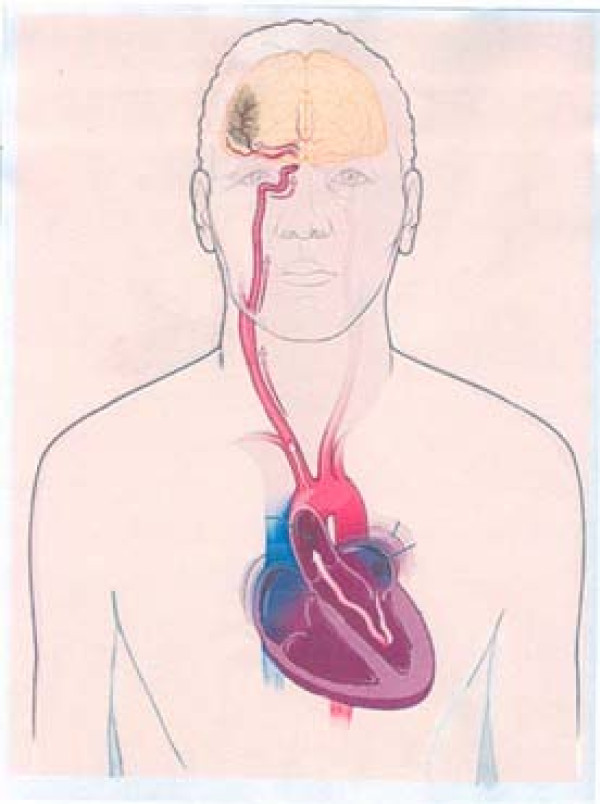
**Picture used to facilitate pathophysiology of stroke in NVAF**.

Using a picture demonstrating the relationship between the brain and the heart taken from the NHLBI patient information website (Figure 2), [14] the research assistant then points out the connection of the heart to the brain:

*"People generally think that heart diseases only cause problems in the chest. But this picture shows you why atrial fibrillation can cause a stroke. As you can see the heart is connected to the brain by a big blood vessel. Afib can cause blood clots in the heart. These clots can travel up the blood vessel to the brain and cut off the blood supply to a part of the brain. This causes a stroke"*.

### Presentation of Individualized Treatment Choices and Outcomes

In NVAF, the benefits (i.e. stroke risk reduction) and harms (i.e. increased bleeding risk) of treatment vary considerably, such that the benefit to harm ratio can actually reverse according to the patients' specific comorbid conditions [6]. In addition, age and comorbidity confer a baseline bleeding risk, [15] which, if not accounted for, results in an overestimation of the bleeding risk associated with treatment. The varying likelihoods of benefits and harms highlights the need for providing patients with individualized risk estimates, calculated separately for bleeding and stroke risk.

We searched for validated risk calculators that would allow us to present patients with individualized outcome data. Of the two calculators identified for baseline stroke risk in NVAF, [4,16] we elected to use the CHADS_2 _algorithm (**c**ongestive heart failure, **h**ypertension, **a**ge, **d**iabetes, **s**troke) because it was developed in a cohort with prevalent rather than incident NVAF. The relative reduction in stroke risk associated with aspirin (21%) and with warfarin (67%) as compared to no treatment were taken from a meta-analysis of randomized controlled trials [1]. Of the number of well-validated calculators for bleeding risk associated with warfarin, [5,17,18] we elected to use the HEMORR_2_HAGES score [5] (**h**epatic or renal disease, **e**thanol abuse, **m**alignancy, **o**lder age, **r**educed platelet count or function, **h**ypertension [uncontrolled], **a**nemia, **g**enetic factors, **e**xcessive fall risk, and **s**troke) because it was derived from population-level data, as compared to data derived from single-site studies, and provides the greatest the greatest range of bleeding risk, with the ability to discriminate risks within this range.

There were surprisingly little data regarding absolute rates of baseline bleeds and risk factors associated with baseline and aspirin associated bleeding risk. Although observational studies of NSAID-associated bleeding frequently include additional bleeding risk factors, the control groups for these studies generally include persons who are taking aspirin, so that they cannot provide an estimate of baseline bleeding risk. The algorithm for baseline bleeding risk stratification was based on meta-analysis of studies in which aspirin use was well characterized [15]. A literature search failed to turn up risk prediction tools for bleeding on aspirin and revealed a wide range of estimates for aspirin's effect on bleeding risk. The most comprehensive systematic review of 17 epidemiologic (observational) studies provided a pooled relative risk for aspirin-associated bleeding of 2.6, but noted that the individual relative risk estimates were heterogeneous, ranging from 1.4 to 11.2 [19]. This review reported that the risk associated with aspirin was not modified by age or gender. Meta-analysis of randomized controlled trial data reports a pooled relative risk for aspirin-associated bleeding of 1.6, with a range from 1.2 to 2.0 [20]. We elected to use a relative risk of 2 for aspirin-associated bleeding risk because it came from the same meta-analysis providing baseline bleeding risk.

**Figure 3:**
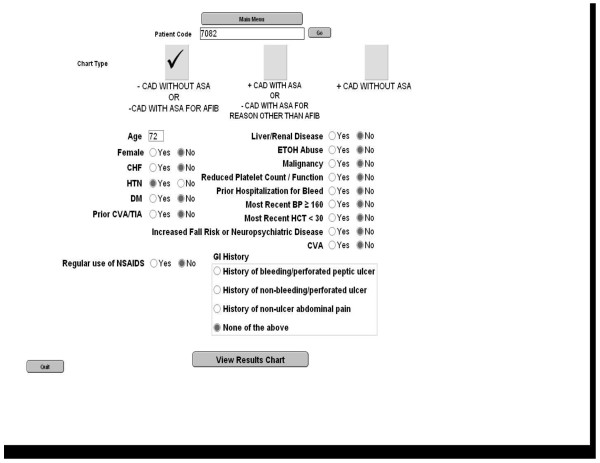
**Data entry screen**.

The risk calculators utilized the "person-year" outcome, which is most commonly interpreted as providing one-year outcomes. However, one-year risks are sufficiently low and may lead to patients undervaluing these outcomes [21]. We therefore converted annualized outcome rates to five-year risks using the declining exponential approximation of life expectancy [22]. FileMaker Pro 9 Advanced (FileMaker, Inc, Santa Clara, CA) was used to create a program that calculated and displayed these outcome rates. The patient's risk factors necessary to calculate the risk of stroke and bleeding for each of the treatment options are entered into a data entry screen, prior to the encounter with the patient (Figure 3).

Although warfarin has been shown to decrease myocardial infarction risk, [23] at our center, many patients with coronary artery disease are prescribed aspirin, regardless of whether or not they are taking warfarin. To ensure that options presented were consistent with local practice norms the tool was designed to show patients the treatment options available to them depending on whether or not they have coexisting coronary artery disease. The addition of aspirin to warfarin was assumed to confer no additional stroke risk reduction as compared to warfarin alone.

**Figure 4:**
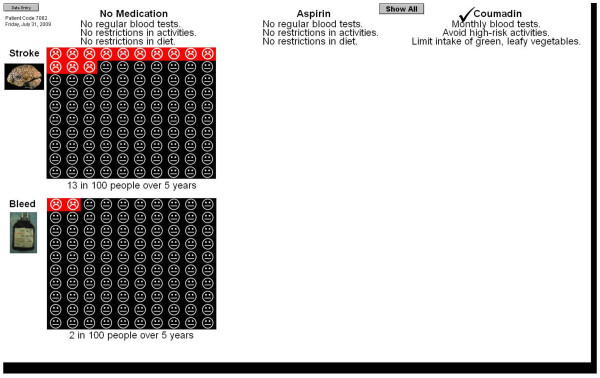
**Presentation of outcome data for single treatment option**.

Patients without coronary artery disease are presented with the options of no anticoagulation, aspirin alone, and warfarin alone (Figure 4). According to their individual comorbidities, patients' 5-year risk of stroke with no therapy potentially ranges from 9 to 60%, with aspirin from 7 to 51%, and with warfarin from 3 to 26%. The 5-year risk of bleeding with warfarin potentially ranges from 9 to 46%, with aspirin from 1 to 45%, and with no therapy from 1 to 26%. Patients with coronary artery disease who are currently taking aspirin are presented with the options of aspirin alone and aspirin plus warfarin. Patients with coronary artery disease who are taking warfarin alone are presented with the options of aspirin alone and warfarin alone.

The practical issues related to each of the treatment choices are also presented. No treatment and aspirin are described as being associated with no need for regular blood tests and no restriction of activities or diet. Warfarin is described as requiring monthly blood tests, avoidance of activities that can cause serious injury, and watching the amount of green, leafy vegetables in the diet.

In addition, given that what matters most to patients are not the risks of stroke or bleed per se, but the sequelae of these, [24,25] we identified data describing the outcome of stroke and major bleed in terms of mortality and function [26,27].

"When people hear the word stroke, they might imagine someone who can't walk or who is in a nursing home. This isn't always the case. We can't know in advance how bad a stroke will be, but in general we know that about half of all people who have a stroke will recover, one-quarter will be disabled, and one-quarter will die."

"There are 2 types of major bleeding. These can happen inside your stomach or your brain. Patients with major bleeding need to be hospitalized and have blood transfusions. We can't know in advance how bad a bleed will be, but in general we know that about three-quarters of all people who have a bleed will recover, and a small number will be disabled or die."

**Figure 5:**
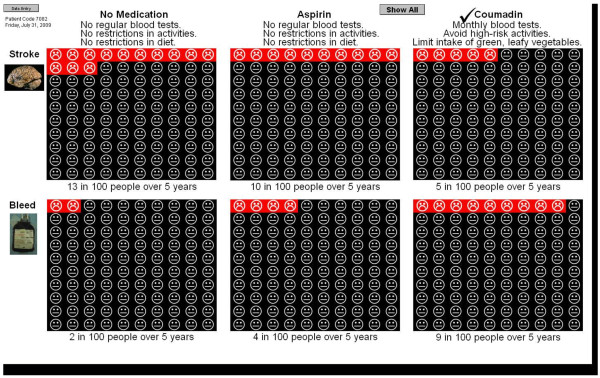
**Presentation of outcome data for all treatment options**.

To enable patients to consider the incremental risks and benefits associated with each option, pictographs illustrating the expected number of strokes and major bleeds with each option are shown sequentially. Patients are then provided the opportunity to view all available options on the same screen (Figure 5).

### Empowering Patients to Understand the Importance of their Participation

Helping patients to understand *how *their participation results in improved treatment decisions can serve to increase their awareness of the benefits of, and their motivation for, engaging in informed choice. The challenge for decision tools is to help patients understand the role of their values and preferences in the process of decision making while acknowledging that many patients do not want to be the primary decision maker. To balance the need to help patients understand that the "best" treatment for NVAF depends on each patient's values with the recognition that many, especially older, persons, rely on their physician's recommendations and do not want to make the final treatment decision, [28] we emphasized that the goal of the tool was for both patients and physicians to have a clear of understanding of how individual patients value the outcomes related to each treatment alternative:

*"So as you can see, there is no perfect medication for atrial fibrillation*.

Because there is no perfect medication, there is no single best choice for everyone. Each person will have different opinions about the right balance between a medication's good effects and a medication's bad effects. You need to think about how important it is to you to lower your risk of a stroke compared to the risk of having a major bleed and the hassle of taking coumadin."

"It is important for your doctor to know how you feel about these treatments. It will be important for you to talk with your doctor about your concerns, questions, and opinions. Your doctor wants and needs to know your thoughts about this, so that he/she can make the best possible recommendation for you."

"Talking about what's important to you will help your doctor make better treatment recommendations for you."

### Developing Patients' Communication Skills

The NVAF tool is built on the recognition that the final treatment choice is best made through a process of patient-clinician communication. It is designed to promote patients' communication skills by including multiple opportunities for them to engage in conversation with the facilitator throughout the tool [11]. The tool opens with a screen consisting of a series of photographs of patients and physicians talking to one another. Participants are asked to describe what they are seeing in the photographs, while the accompanying script highlights both the importance and challenges of good patient-physician communication:

"*How well you and your doctor talk to each other is one of the most important parts of getting good health care. But sometimes talking to your doctor is not easy because you're not sure what to say."*

The tool also asks patients to think about each treatment option by discussing their views on the benefits and harms. This approach helps patients to clarify their values in the context of practicing what they may want to communicate to their physician. If they have difficulty answering, the research assistant encourages them by saying:

"Let's think about them one at a time. What are your feelings about aspirin? What do you like about that as a treatment and what do you dislike?"

The final section directly prepares participants for communicating with their physicians by asking them to write down specific questions they would like to ask in the form of a worksheet.

"Let's think about what questions or comments you might have for your doctor. I'd like you to write them down here."

### Providing Patients with the Opportunity to Participate

There is ample evidence in the literature that patients, including those that perceive themselves as empowered, frequently fail to attain their desired level of participation [29]. Some patients do not have effective communication skills while others may simply not be afforded the opportunity [30]. These findings support the need for decision support tools to target physician, as well as patient, communication. In order to increase the probability that patients are given the opportunity to participate, the tool includes a prompt for physicians to use during the encounter:

"I know you just learned about your treatment options for your Afib. Can you tell me how you feel about them?"

The prompt, which is handed to the physician by the facilitator as the patient enters the examination room for the clinical visit, was created to ensure that the decision support tool exert an effect on the "real-time" clinical interaction between doctor and patient. This interaction serves several purposes. For example, the clinician can assess patient's knowledge regarding different treatment options and address any misperceptions the patient may have. With their knowledge of the individual patient's clinical and psychosocial status, clinicians can help patients to understand better how his/her values and preferences can best inform decision making.

An alternative approach to providing patients with the opportunity to participate is to create entire tools for use by the physician within the clinical visit. However, physicians' time constraints make it difficult to routinely include these tools in clinical practice. The proposed model, and design of the NVAF tool, is consistent with the goals and organization of the patient-centered medical home in which all members of the patient's healthcare team utilize their top skill sets [31]. The initial part of the tool, focusing on patient preparation, can be administered by a nurse or other mid-level practitioner. The completion of the worksheet, along with a specific physician prompt, are designed to extend the reach of the tool, both literally and figuratively, into the clinical encounter during which time the physician can actively engage the informed and prepared patient in the decision making process.

### Feasibility and Acceptability

The computer tool underwent testing for feasibility and acceptability with participants identified as having had a primary care visit at the VA Connecticut Healthcare System with a diagnosis code for atrial fibrillation within the last 24 months. Charts were reviewed to confirm the diagnosis of NVAF and for exclusion criteria, which included: a) diagnosis of dementia, b) legal blindness, and c) contraindication to warfarin or aspirin. The clinicians for potential participants were also asked to indicate whether their patients met any of the following exclusion criteria: a) atrial fibrillation managed by a physician outside of the VA, b) the patient would not be able to understand numeric data based on cognitive impairment, c) the patient had a life expectancy of < 12 months. Participants were also excluded if they failed a clock-drawing task performed at the start of their study participation.

Eligible participants met with a trained research nurse immediately prior to a regularly scheduled primary care visit to complete the computer tool. Just after completing the tool, participants were asked about its ease of use and acceptability.

A total of 11 participants used the tool. Participants had mean age of 78 ± 9 years (range 61-90), and 7 (64%) had completed ≤ 12 years of education. The tool took between 20 and 35 minutes to administer. On a scale from 1 to 5, ranging from very easy to very hard, 8 (72%) rated the ease of completing the tool as a 1, with no participant rating the ease of the tool as > 3. When asked about the amount of information provided by the tool on a scale of 1 to 5, with 1 = "too little," 3 = "just right," and 5 = "too much," 9 (81%) participants rated the amount as 3, and 2 participants rated the amount as 2. When asked if they would recommend the tool to others, 10 (91%) responded "yes."

## Discussion

The development of a new decision support tool for anticoagulation for NVAF was based on the desire to provide patients with individualized outcome data regarding their risks of stroke and bleeding associated with all reasonable treatment options. It sought to provide these data in the context of preparing patients to participate in the decision-making process with their physicians by helping them to understand why treatment of NVAF involves a choice and the role of their values in the decision and by encouraging patient-physician communication. In pilot testing, the majority of participants reported that the tool was easy to complete and contained the right amount of information, and that they would recommend the tool to a friend.

Embedded within the tool is a risk calculator which provides patients with individualized outcomes related to all available alternatives over a meaningful time period. Building upon the work of prior tools [9,10,32] to provide patients with individualized outcomes of stroke risk, the tool uses the best available current observational data to individualize bleeding risk according to the patient's specific comorbidity profile. There is wide variability in both stroke and bleeding risk, and, because different comorbidities are associated with stroke and bleeding, the benefit-to-harm ratio can reverse depending upon the particular combination of comorbid conditions [6]. This tool allows patients to consider explicitly the trade-offs between the reduction in stroke risk versus increase in bleeding risk associated with different treatment options, with each of these risks separately calculated for each individual patient. In addition, we provide patients with an explicit description of the sequelae associated with both stroke and major bleed.

The tool also helps patients to understand the importance of their values in decision making for NVAF. The language we employed is careful to reflect the finding that almost all patients count on their physicians' recommendations and many patients may not want to make treatment decisions for themselves [28,33]. The objective is to have patients communicate their views regarding the trade-offs among the different treatment options to their physicians, so that a decision can be made according to the preferred decision-making style of the patient, which may be to share in making the decision or to have the physician make the decision. Addressing the quality of physician-patient communication requires intervening both on the part of the patient and the physician. The tool is built on the recognition that patients' communication styles can have a substantial influence on physicians' behavior [34] and that, through intervention aimed at preparing patients with appropriate questions, patients' communication skills can be improved such that they give more information to and receive more information from their physicians[35]. The tool also acknowledges that patients, including those that perceive themselves as empowered, frequently fail to attain their desired level of participation, [36,37] by including a physician prompt that invites patient communication.

There are several limitations in the individualized risk information the tool provides. First, both the CHADS_2 _and HEMORR_2_HAGES scores were based on Medicare data, and their validity for use among younger patients has not been established. Second, the relative risk of bleeding with aspirin is assumed to be constant across all individuals, which does not allow for the possibility of interactions between aspirin and bleeding risk factors. Moreover, we did not address the known interactions between warfarin and other medications, such as NSAIDs. Third, although we elected not to use randomized controlled trial data to describe the probability of intracranial versus extracranial bleeding, the differences in mortality and functional sequelae of these two outcomes suggest that they should be presented as two distinct outcomes. Finally, it is likely that the bleeding risk associated with warfarin decreases over time,[38] but we did not have sufficient data to calculate a differential bleeding risk over time.

## Conclusions

Building upon the work of earlier tools, this new decision support tool provides individualized outcome data for stroke and bleeding risk associated with all appropriate treatment options, and it incorporates elements designed to promote physician-patient communication. Additional research is necessary to determine the effect of the tool on knowledge and decisional conflict, outcomes routinely included in and improved by decisions support tools [39]. The tool has also been designed to achieve the goal of improving the quality of decision-making for NVAF by ensuring that the decision incorporates the informed patient's values. This outcome has not frequently been included in studies of decision support tools [39]. The challenge for future intervention trials is how to measure this outcome, which depends upon the complex interplay of the patient's understanding of his/her illness, the patient's values and preferences, and the opinions and preferences of the physician.

## Competing interests

The authors declare that they have no competing interests.

## Authors' contributions

All authors read and approved the final manuscript. All three authors contributed to the study concept and design, acquisition of data, analysis and interpretation of data, and preparation of manuscript.

## Pre-publication history

The pre-publication history for this paper can be accessed here:

http://www.biomedcentral.com/1472-6947/11/59/prepub
